# Infection by Wolbachia bacteria and its influence on the reproduction of the stored-product psocid, Liposcelis tricolor

**DOI:** 10.1673/2006_06_24.1

**Published:** 2006-09-28

**Authors:** Peng Dong, Jin-Jun Wang, Zhi-Mo Zhao

**Affiliations:** Key Laboratory of Entomology and Pest Control Engineering, College of Plant Protection, Southwest University, Chongqing 400716, P. R. China

**Keywords:** endosymbionts, molecular detection, antibiotic treatment

## Abstract

Wolbachia are maternally inherited intracellular bacteria that infect a wide range of arthropods and nematodes and are associated with various reproductive abnormalities in their hosts. The infection by Wolbachia of the psocid, Liposcelis tricolor (Psocoptera: Liposcelididae), was investigated using long PCR amplification of the *wsp* gene that codes for a Wolbachia surface protein. The results showed that L. tricolor was positive for Wolbachia. Phylogenetic analysis showed that the Wolbachia found in L. tricolor was related to the B-group. Wolbachia infection in L. tricolor could be removed through antibiotic treatment. The results of crosses including ♀^W+^ x ♂ ^W+^, ♀ ^W−^ x ♂^W+^, ♀ ^W+^ x ♂ ^W−^, and ♀^W−^ x ♂ ^W−^, suggested that the removal of Wolbachia resulted in lower egg production by L. tricolor. The mean embryonic mortality of offspring produced by L. tricolor without Wolbachia was significantly higher than that of control.

## Introduction

 Wolbachia, rickettsia-like proteobacteria, are found in the reproductive tissues of a wide range of arthropod species (O’Neill et al. 1997; Werren 1997; Tram and Sullivan 2002). Wolbachia are obligate intracellular parasites that are transmitted maternally from infected females to their progeny. Wolbachia infection are associated with a variety of reproductive anomalies in the host, including cytoplasmic incompatibility between different populations (Laven 1967) or closely related species (Breeuwer and Werren 1990; Breeuwer et al. 1992), thelytoky or parthenogenesis induction in parasitoid wasps (Stouthamer et al. 1990; 1993), male-killing in coccinellid beetles (Hurst et al. 1999), and feminization of genetic males in isopods (Rousset et al. 1992).

 Wolbachia cannot be cultured in defined media and detection within infected gonadal cells may be time consuming. Therefore, detection of Wolbachia infection has been based largely on amplification of Wolbachia DNA using allele-specific polymerase chain reactions. Primers designed from the 16S rDNA (O’Neill et al. 1992), ftsZ (Werren et al. 1995) and wsp genes (Zhou et al. 1998; Jeyaprakash and Hoy 2000) have been used to amplify Wolbachia DNA from a diverse array of arthropods.

Antibiotic treatment is one established method for producing Wolbachia-free individuals (O’Neill and Karr 1990; Yusuf and Turner 2004). A wide range of antibiotics with different modes of action have been used to produce aposymbiotic insects (Stouthamer et al. 1993). The strains of parthenogenetic Trichogramma wasps revert to sexual reproduction when rickettsial endosymbionts are eliminated from wasp gonads (Stouthamer et al. 1993).

The psocid, Liposcelis tricolor, a bisexual species, is worldwide and commonly found in various processed and unprocessed dry foods in households, granaries, and warehouses (Kalinovi and Rozman 2000). They transmit harmful microorganisms, including fungi and bacteria on their bodies, hair, and in feces. Psocids are found to be harmful pests on seed goods as well by causing damage to seed kernels (Kalinovi and Rozman 2000). This study was initiated to find if Wolbachia infest L. tricolor and their influence on the reproductive biology of L. tricolor.

## Materials and Methods

### Insects

Stock colonies of Liposcelis tricolor originated from larva collected in a wheat warehouse in Shandong, China in 2003. This colony was reared on an artificial diet consisting of whole wheat flour, skim milk and yeast powder (10:1:1) in an air-conditioned room at 27±1 °C and a scotoperiod of 24 h. Cultures were set up in glass bottles (250 ml) with a nylon screen cover and kept in desiccators (5 liter), in which the humidity was controlled with saturated NaCl solution at 75–80% RH. After several generations, insects from the stock colony were used for the tests.

### DNA extraction

Twenty adult L. tricolor were surface-sterilized in a series of double distilled water and 70% ethanol washes, then frozen under liquid nitrogen and crushed in 300 μl DNA extraction buffer (100 mM Tris-HCl, pH 8.0, 50 mM NaCl, 50 mM EDTA, 1% SDS, 0.15 mM spermine, 0.5 mM spermidine), homogenized using a DNA-free disposable polypropylene pestle and incubated with 2 μl of proteinase-K (20 mg/L) for 2h at 50° C, followed by 5 min at 95 °C for denaturing. One volume of phenol saturated water (pH8.0) and 1 volume of chloroform:isoamyl alcohol (24:1) was added before centrifugation for 10 min at 10000 rpm. The supernatant was collected and gently mixed with 0.2 volume of Na-acetate (3 mM, pH5.2) and 2 volumes of 100% ethanol. After precipitation for 2h at −20 °C, the DNA was washed with 70% ethanol, air dried and finally resuspended in 20 μl of ddH_2_O. 3 μl of the DNA sample was used for PCR experiments. DNA quality was determined using insect 12S rDNA primers. The PCR mixtures and cycling conditions were from Yoshizawa and Johnson (2003).

### PCR screening procedure for Wolbachia infection

Long PCR was performed in a 25 μl containing 50 mM Tris (pH 9.2), 16mM ammonium sulphate, 1.75 mM MgCl_2_, 350 μM dNTPs, 800 pM of primers (wsp-F, 5′-TGG TCC AAT AAG TGA TGA AAG AAA CTA GCT A and wsp-R, 5′-AAA AAT TAA ACG CTA CTC CAG CTT CTG CAC), 1 unit of *Pwo* and 5 units of *Taq* DNA polymerases. The Long PCR was carried out using three linked profiles: (i) one cycle (2min at 94 °C), (ii) 10 cycles (10s at 94 °C, 30s at 65° C, 1min at 68 °C), (iii) 25 cycles (10s at 94 °C, 30s at 65 °C, 1 min at 68 °C, plus an additional 20s added for every consecutive cycle).

To confirm that the PCR products obtained were not due to contamination, an attempt was made to sequence the long PCR products directly from L. tricolor. The PCR product was then ligated into a pGEM-T vector. Three clones were produced that were sequenced by Shanghai Sangon Biological Engineering Technology & Services Company.

### Phylogenetic analysis

The wsp datasets representative sequences for Wolbachia groups in different hosts were retrieved from GenBank and included in phylogeny reconstruction. Sequences were aligned using CLUSTAL W (Thompson et al. 1994). Phylogenies for wsp were estimated by neighbor-joining analysis of the sequence data. Gaps were coded as missing data. Distances were calculated using maximum likelihood. The model of sequence evolution and the parameter values best fitting the data for each locus were identified using likelihood ratio tests (Huelsenbeck and Pannala 1997), as implemented in PHYLIP 3.57c (Felsenstein 1995) to search for the tree with the highest likelihood. The parameter values suggested by PHYLIP for each dataset were used to specify the distance matrix for neighbor-joining analysis on 1000 bootstrap replicates.

### Antibiotic experiments

Rifampicin, a potent inhibitor of DNA-dependent RNA polymerase of bacteria, was used to produce Wolbachia-free hosts. Rifampicin (1%) was prepared by mixing 10g of the standard diet with 0.1 g rifampicin (Sigma, www.sigmaaldrich.com). Three grams of this food-antibiotic mixture were further diluted into 10 and 100g of standard diet to make final concentrations of 1%, 0.3%, and 0.03% antibiotic in the standard diet. Dry mixing of the food and antibiotic powder proved unsatisfactory and instead the food-antibiotic mixture was moistened with sterile distilled water, well stirred to create homogeneous slurry, air-dried and ground to a powder (Yusuf and Turner 2004). After 4 weeks treatment with rifampicin, the psocids were checked with the above molecular method. After two generations, the psocids of Wolbachia-free strain (W^−^) and untreated stain (W^+^) were used for the following cross experiment.

### Effects of Wolbachia removal on fecundity and fertility

Four crosses (♀ ^W+^ x ♂^W+^, ♀ ^W−^ x ♂ ^W+^, ♀ ^W+^ x ♂ ^W−^, and ♀^W−^ x ♂ ^W−^) were tested in this experiment. The cross of the infected female and male was used as a control. In each cross experiment, 30–61 maturing females (when the colorless stage begins to change to brown, 12–24 h after the final molt) were removed from culture populations with a fine brush. They were placed individually in a small glass vials containing a piece of filter paper on one side of which was glued the culture medium. The glass vials were covered with fine mesh net to prevent psocids escape. One corresponding matured male was placed into each glass vials. The glass vials were placed in each humidified desiccator (75–80%RH). The desiccators were placed in growth chambers at 27.5 °C with a scotoperiod of 24 h. Eggs produced from the different treatment were counted, and the mortality of eggs from the different treatments was monitored. The total egg numbers within 60 days were counted for each experiment, and the statistical analysis was conducted using SPSS statistical package.

## Results

**Figure 1:**
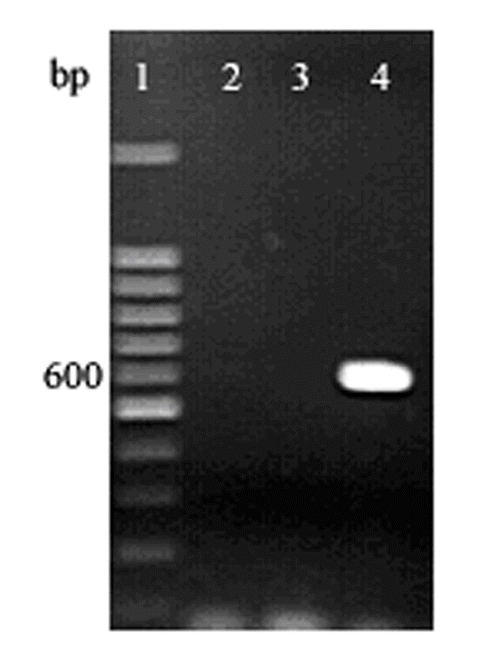
Long PCR amplified wsp sequences from L. tricolor. Lane 1, 100bp DNA marker; lane 2 and 3 no DNA-control; lane 4, L. tricolor

Genomic DNA prepared from L. tricolor, was amplified by Long PCR, using the primers (wsp-F and wsp-R). A 605 bp fragment of the wsp gene was amplified (Fig.1) and this sequence was deposited in GenBank with accession number AY639593. The infection frequency of Wolbachia in L. tricolor was found to be 100% in 5 randomly selected females and 5 males.

**Figure 2:**
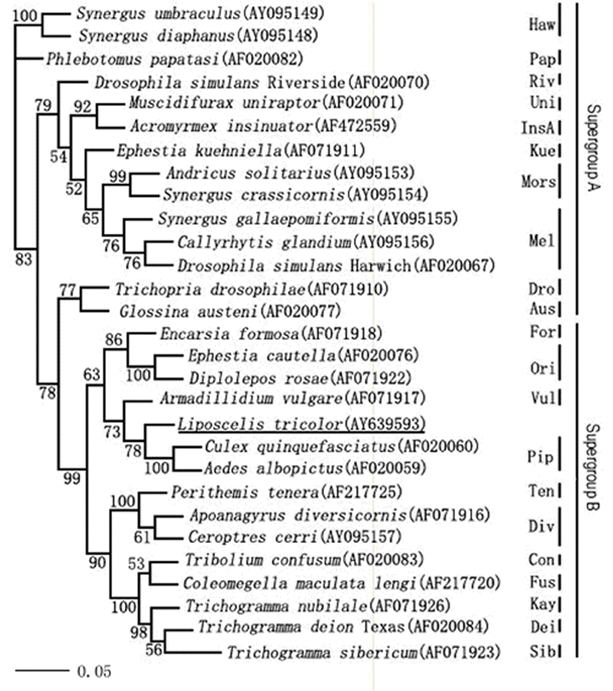
The phylogenetic relationship between the different Wolbachia wsp sequences as reconstructed using a neighbour-joining algorithm on maximum likelihood estimated distance matrix. Name of the host arthropod species is followed by GenBank Accession no. and clade designation. The Wolbachia groups to which sequences belong to are also shown. Bootstrap values of 1000 replicates are presented above the branches. Bootstrap values less than 50 are not shown. Wolbachia in L. tricolor was shown by underling of group.

The phylogenetic tree based on the wsp sequences using neighbor-joining after bootstrapping 1000 times is shown in Fig. 2. The topology showed the division of Wolbachia into two subgroups, A and B. The A subgroup included fourteen species and the B subgroup included fifteen species. Wolbachia in L. tricolor belonged to the B subgroup and was close to Wolbachia Pip in Culex quinquefasciatus and Aedes albopictus.

**Figure 3:**
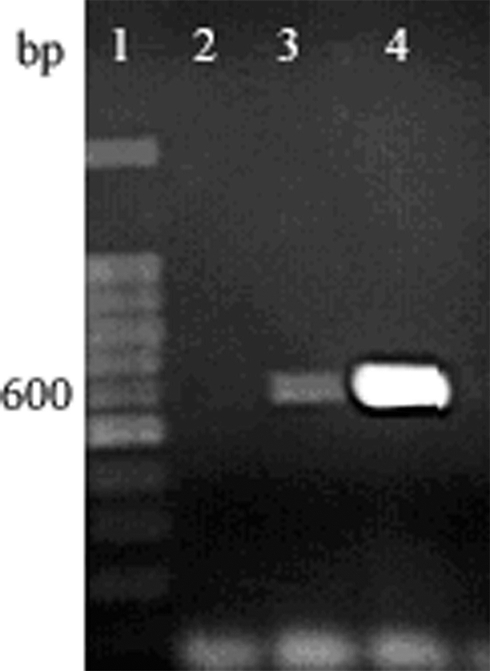
Long PCR amplification of wsp sequences from L. tricolor treated with antibiotic of different dosages. Lane 1, 100bp DNA marker; Lane 2, 1% rifampicin; lane 3, 0.3% rifampicin; lane 4, 0.03% rifampicin.

After 4 weeks treatment with 1% rifampicin, no detectable level of the wsp gene was amplified, while the same gene fragment remained present in the 0.3% or 0.03% rifampicin treated individuals (Fig. 3). A strain of L. tricolor without Wolbachia infection was obtained.

**Figure 4:**
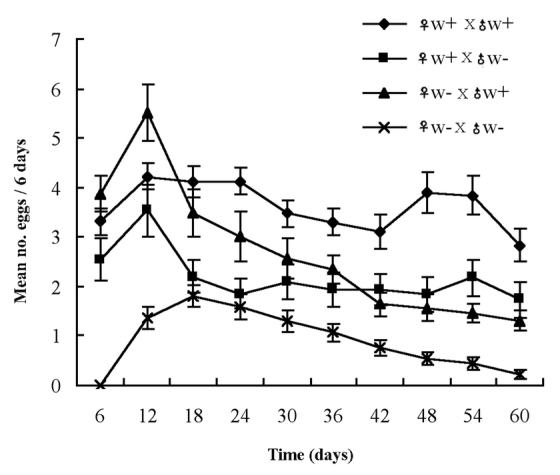
Effects of Wolbachia on fecundity and fertility of L. tricolor.

In cross experiments, all combinations could produce eggs. Total egg production was significantly increased in crosses with Wolbachia present, and egg mortality was significantly lower (Table 1, Fig. 4).

## Discussion

In preliminary experiments both regular and long PCR protocols were used to detect the infection of Wolbachia in L. tricolor in the present study, and the target fragment was scored in both protocols. However, there were some non-specific fragments that appeared using the regular PCR protocol. Therefore, the long PCR protocol was used throughout the experiment.

Phylogenetic evidence has shown that horizontal transfer of Wolbachia must have occurred in the course of evolution because closely related bacterial strains can be found in unrelated hosts (Huigens et al. 2004; O’Neill et al. 1992; Werren and Zhang 1995). In this study, phylogenetic analysis showed that Wolbachia in L. tricolor was close to *w*Pip present in Culex quinquefasciatus and Aedes albopictus.

In the present study, a strain of L. tricolor without Wolbachia infection was obtained by treating with 1% rifampicin for 4 weeks. Lower concentrations of rifampicin (0.3% or 0.03%) failed to remove the Wolbachia infection completely. Similar results were reported for tetracycline treatment of Wolbachia infection in Aedes bopictus (Dobson and Rattanadechakual 2001) and rifampicin treatement of Liposcelis bostrychophila by Yusuf and Turner (2004).

 Wolbachia have a variety of different effects on their hosts’ reproduction. Cytoplasmic incompatibility is the most widespread and, perhaps, the most prominent feature that Wolbachia endosymbionts impose on their hosts. Cytoplasmic incompatibility resulted in embryonic mortality in crosses between insects with different Wolbachia infection status. It can be either unidirectional or bi-directional. Unidirectional cytoplasmic incompatibility is typically expressed when an infected male is mated with an uninfected female (Hoffmann and Turelli 1997; Charlat et al. 2001). However, all cross combinations in this study could produce hatching eggs, and the cross ♀^w^^−^ x ♂^w+^ did not show higher embryonic mortality compared to the ♀^w^^−^ x ♂^w^^−^ cross. As a result there is not enough evidence to confirm cytoplasmic incompatibility phenomenon caused by Wolbachia in L. tricolor.

 Wolbachia could have positive effects on host fecundity (Girin and Bouletreau 1995; Stolk and Stouthamer 1996) and fertility (Wade and Chang 1995). In this study, egg production of ♀^w^^−^ mated with ♂^w^^−^, ♀^w+^ with ♂^w^^−^ and ♀^w^^−^ with ♂^w+^ were all significantly less than the control. The eggs with the highest survival were from the control crosses with both male and female infected, and the other crosses are statistically distinguishable. This suggests that for optimal reproduction, Wolbachia must also be present in the male. It implies that Wolbachia infection can have positive effects on fecundity and fertility of L. tricolor. Similarly, Dedeine et al. (2001) reported that the wasp Asobara tabida without Wolbachia removed by antibiotics also had lower egg production. By comparison, the mortality of eggs produced by L. tricolor without Wolbachia infection was significantly increased. This could be due to cooperative evolution between Wolbachia and the psocid host.

In recent years, there has been increasing interest in the biology of Wolbachia and in its application as an agent for control or modification of insect population (Zabalou et al. 2004). Therefore, the mode of action of Wolbachia manipulation on psocid reproduction warrants further investigation.
